# Identification of Patients With Locally Advanced Gastric Cancer Who May Benefit From Adjuvant Chemoradiotherapy After D2 dissection: A Propensity Score Matching Analysis

**DOI:** 10.3389/fonc.2021.648978

**Published:** 2021-04-01

**Authors:** Shu-Bei Wang, Wei-Xiang Qi, Jia-Yi Chen, Cheng Xu, Wei-Guo Cao, Rong Cai, Lu Cao, Gang Cai

**Affiliations:** Department of Radiation Oncology, Ruijin Hospital, Shanghai Jiaotong University School of Medicine, Shanghai, China

**Keywords:** chemoradiotherapy, propensity score matching, para-aortic lymph nodes, patient selection, gastric cancer

## Abstract

**Background:**

One of the most controversial areas in gastrointestinal oncology is the benefit of postoperative chemoradiotherapy (CRT) over chemotherapy (CT) alone after D2 dissection of locally advanced gastric cancer (LAGC). We aimed to identify the LAGC patients who may benefit from adjuvant CRT.

**Methods:**

We analyzed retrospectively 188 patients receiving radical gastrectomy with D2 dissection for LAGC in our hospital. Patients were divided into two balanced groups by using propensity score matching: CRT group (n = 94) received adjuvant CRT, and CT group received adjuvant CT alone.

**Results:**

At a median follow-up of 27.10 months, 188 patients developed 79 first recurrence events (36 in CRT group and 43 in CT group). Our results showed that adjuvant CRT significantly decreased the risk of developing local regional recurrence (LRR) when compared to CT alone (14.9% *vs.* 25.5%, *p* = 0.044), while the estimated 3-year disease-free survival (DFS) was comparable between the CRT and CT groups (59.3% *vs.* 50.9%, *p* = 0.239). In the subgroup analysis, a significantly decreased LRR rate was also observed in LAGC patients with N1-3a stage after adjuvant CRT (*p* = 0.046), but not for N3b. Para-aortic lymph nodes (station No. 16) were the most frequent sites of LRR. After receiving radiotherapy, recurrence of 16 a2 region and 16 b1 region were significantly deceased (*p* = 0.026 and *p* = 0.044, respectively). Patients who received irradiation more than 4 months after surgery showed an increased risk of LRR (*p* = 0.022).

**Conclusions:**

This study showed that adjuvant CRT significantly reduced LRR after D2 dissection of LAGC. Early initiation of adjuvant RT with clinical target volume encompassing a2 and b1 regions of para-aortic lymph nodes is recommended for pN1-3a patients after D2 dissection.

## Introduction

Gastric cancer remains a major cause of cancer death in China and globally (1, 2). Most patients with gastric cancer in China are symptomatic and diagnosed with locally advanced disease (3). The positive impact of adjuvant (postoperative) and neoadjuvant (preoperative) strategies on survival in patients with resected gastric cancer has become clearer over time, although there is no consensus as to the best approach.

One of the most controversial areas in gastrointestinal oncology is the benefit of postoperative chemoradiotherapy (CRT) over chemotherapy (CT) alone after D2 dissection of gastric cancer. The treatment failure in patients with resected gastric carcinoma is related to the loco-regional recurrence (LRR) at some points (4). However, despite the multiple randomized trials and meta-analyses, the survival benefit from adding CRT to CT after D2 dissection of gastric cancer remains uncertain.

The ESMO guidelines suggest that for patients with stage ≥IB gastric cancer who have undergone surgery without neoadjuvant CT, either postoperative CRT or adjuvant CT is appropriate (5). On the other hand, guidelines from the NCCN suggest postoperative CRT plus CT for patients with pathologic T3-4 or node-positive disease if they have undergone less than a D2 dissection, and CT alone for those who have undergone a D2 lymph node dissection (6).

Thus, in this study, we retrospectively analyzed patients with gastric cancer who received adjuvant CRT after D2 lymph node dissection in comparison with those who received CT alone. The potential stratification factor for patient selection is the second question of our study. To minimize confounding factors, propensity score matching was used in this study.

## Materials and Methods

### Patients’ Characteristics

We retrospectively identified consecutive patients who were treated between January 2010 and December 2018 in our hospital. The inclusion criteria were as follows: 1) underwent radical gastrectomy (R0 resection) with D2 lymph node dissection, at least 15 lymph nodes dissected; 2) histologically confirmed gastric adenocarcinoma, with no clinical evidence of distant metastasis (M0), according to the American Joint Committee on Cancer (AJCC) eighth edition (7); 3) no history of any other cancer or previous radiotherapy or CT, or coexisting malignancies.

### Radiotherapy

Radiotherapy was delivered by a linear accelerator with 6 MV photon beam based intensity modulated radiation therapy (IMRT). Patients were irradiated with a total dose of 41.4 to 54 Gy (median, 45 Gy), with 1.8 to 2 Gy daily fractions administered over 4 to 6 weeks. The clinical target volume included tumor bed, anastomotic stoma, and regional draining lymph nodes. The tumor bed and anastomotic stoma were determined by preoperative and postoperative Computed Tomography (CT) imaging and surgical clips. Regional lymph nodes included perigastric, celiac, splenic, hepatoduodenal or hepatic-portal, and panceraticoduodenal depending on the location of the tumor (8). For all the patients, the para-aortic nodes were irradiated. The planning target volume (PTV) margin was 0.8 to 1.5 cm, and it was optimized to ensure prescription dose to more than 95% of the PTV. The mean dose of each kidney was < 18 Gy and the mean dose of liver was < 25 Gy. ≤195 cc of bowel to receive >45 Gy. The max dose of spinal cord was ≤ 45 Gy. Treatment plan for radiotherapy was decided by two doctors who have more than 10 years experience in radiation-oncology before the initiation of radiotherapy. Treatment fields, dosimetry, surgery and pathology reports, and preoperative tumor imaging were reviewed before treatment began. The cone beam CT (CBCT) imaging was performed once every week during the radiation therapy treatment.

### Chemotherapy

Concurrent CT regimens were as follows: capecitabine 825 mg/m^2^ bid d1-5 weekly or S-1 40 mg/m^2^ bid d1-5 weekly. All patients received four to eight cycles of Fluorouracil based regimens after surgery. In the CRT group, chemotherapy was delivered in one to three cycles before and three to seven cycles after chemoradiotherapy. In the CT group, patients received four to eight cycles chemotherapy after surgery.

### Data Collection

All patients**’** clinical and pathological characteristics, including age, sex, tumor location, depth of tumor invasion, number of positive lymph nodes, adjuvant treatment, recurrence, and survival information were retrospectively reviewed based on operative notes and medical records.

### Definition of the Recurrence Pattern and Survival Time

All patients were followed up from the date of surgery to death or emigration. The last follow-up of all recurrences was March 2020. The recurrence pattern was determined according to the primary recurrence site diagnosed by imaging scans (CT, US, MRI, or PET-CT), endoscopy, ascitic cytology, or biopsy. The primary recurrence sites in 14 patients were determined by PET-CT (8/43 in CT group and 6/36 in CRT group). Other recurrences were detected by US, CT, or MRI. Local regional recurrence included recurrence at stomas, a duodenal stump, tumor bed, residual stomach and regional lymph nodes. The regions of lymph nodes recurrence were determined according to the Japanese Gastric Cancer Treatment Guidelines (9). Para-aortic lymph node was subgrouped into a1, a2, b1, and b2 (9). Peritoneal dissemination was determined to be any recurrence within the abdominal cavity due to intraperitoneal distribution and mesothelial implantation. Distant metastasis was defined as any metastasis to distant lymph nodes outside the abdominal cavity, distant organs or sites except for the peritoneum (10). Toxicities were assessed using the Common Terminology Criteria for Adverse Effect (CTCAE) 4.0. The DFS was defined as the time from surgery to recurrence or death of any other causes.

### Statistical Analysis

Statistical analyses were performed using SPSS Statistics 19 (IBM Corp., Armonk, NY, USA). Kaplan-Meier analysis and log-rank tests were used to assess DFS and associated factors. A Cox proportional-hazards model was used to detect the potential effective factors for survival. A two tailed *p*-value < 0.05 was considered statistically significant. The propensity score was calculated by using a logistic regression model with a Caliper of 0.01. The propensity score model included age, sex, Bormann type, tumor location, histological differentiation, T stage, N stage, AJCC stage, lymphovascular invasion (LVI), and perineural invasion. We then formed matched pairs between CT and CRT group patients using the nearest neighbor matching method. A nearest neighbor and 1 to 1 matching algorithm was performed within default caliper (0.01) in R version 3.4.2 software (The R Foundation for Statistical Computing, Vienna, Austria. http://www.r-project.org).

## Results

A total of 1,199 patients treated between January 2010 and December 2018 met our criteria. After adjustment for age, T-stage, N-stage, AJCC stage, and tumor location, 188 patients were successfully matched (**Table 1**). Half of the patients (94) received adjuvant CRT after radical surgery and was considered as the CRT group, and other received CT alone and was defined as the CT group. Clinical characteristics of the 188 patients are summarized in **Table 1**. Most of the patients were stage III (>80%) and more than half of them were N3 stage. Patients in the CRT group were irradiated with a median dose of 45 Gy (range, 41.4–54 Gy). In both groups, the median cycle of adjuvant chemotherapy was 6 (4–8). The chemotherapy included 5-FU, capecitabine or S-1, with or without oxaliplatin (71 in CT group and 69 in CRT group) or paclitaxel (19 in CT group and 20 in CRT group).

**Table 1:** Clinicopathological characteristics of 188 gastric cancer patients.

Clinical features	CT group (n = 94)	CRT group (n = 94)	*p*
**Sex, n (%)**			
**Male**	61 (64.9%)	73 (77.7%)	0.053
**Female**	33 (35.1%)	21 (22.3%)	
**Age, median year (range)**	58.5 (28–79)	59 (32–84)	0.689
**Bormann type, n (%)**			
**I**	10 (10.6%)	10 (10.6%)	0.563
**II**	24 (25.5%)	28 (29.8%)	
**III**	54 (57.4%)	52 (55.3%)	
**IV**	6 (6.4%)	4 (4.3%)	
**Tumor location, n (%)**			
**Upper one-third**	14 (14.9%)	19 (20.2%)	0.332
**Middle one-third**	33 (35.1%)	33 (35.1%)	
**Lower one-third**	47 (50.0%)	42 (44.7%)	
**Histological differentiation, n (%)**			
**Well-moderate differentiated tumors**	15 (16.0%)	8 (8.5%)	0.212
**Poorly differentiated and undifferentiated tumors**	79 (84.0%)	89 (91.5%)	
**T stage, n (%)**			
**pT2**	9 (9.6%)	9 (9.6%)	1.000
**pT3**	19 (20.2%)	19 (20.2%)	
**pT4**	66 (70.2%)	66 (70.2%)	
**N stage, n (%)**			
**pN0**	4 (4.3%)	4(4.3%)	1.000
**pN1**	20 (21.3%)	20 (21.3%)	
**pN2**	19 (20.2%)	19 (20.2%)	
**pN3a**	32 (34.0%)	32 (34.0%)	
**pN3b**	19 (20.2%)	19 (20.2%)	
**AJCC stage, n (%)**			
**IA**	0 (0%)	0 (0%)	1.000
**IB**	1 (1.1%)	1 (1.1%)	
**IIA**	3 (3.2%)	3 (3.2%)	
**IIB**	14 (14.9%)	14 (14.9%)	
**IIIA **	29 (30.9%)	28 (29.8%)	
**IIIB**	28 (29.8%)	29 (30.9%)	
**IIIC**	19 (20.2%)	19 (20.2%)	
**LVI, n (%)**			
**Positive**	32 (34.0%)	42 (44.7%)	0.135
**Negative**	62 (66.0%)	52 (55.3%)	
**Perineural invasion, n (%)**			
**Positive**	38 (40.4%)	40 (42.6%)	0.767
**Negative**	56 (59.6%)	54 (57.4%)	

### Side Effects of Chemotherapy and Chemoradiotherapy

Adverse events during postoperative treatment were reported in 80 (85.1%) of CRT patients and 65 (69.1%) of CT patients (*p* = 0.009). **Table 2** shows acute adverse events reported. Toxic reactions in most patients were mild. The CRT patients showed higher frequency of neutropenia (71.3% vs. 54.3%, *p* = 0.016), thrombocytopenia (20.2% vs. 9.6%, *p* = 0.041). However, there was no difference in the incidence of severe toxicity between these two groups. Only 15 patients (8.5% in CRT group and 7.4% in CT group, *p* = 0.788) were at CTCAE grade 3 to 4. The most common grade 3 to 4 toxicities were neutropenia and thrombocytopenia. There were no severe long-term toxic reactions or treatment-related deaths.

**Table 2:** Acute toxicity effects.

	All grades, n (%)			Grade 3 or 4, n (%)		
	CRT	CT	*p*-value	CRT	CT	*p*-value
**Any adverse event**	80 (85.1%)	65 (69.1%)	0.009	8 (8.5%)	7 (7.4%)	0.788
**Neutropenia**	67 (71.3%)	51 (54.3%)	0.016	4 (4.3%)	2 (2.1%)	0.678
**Anemia**	21 (22.3%)	19 (20.2%)	0.722	0 (0.0%)	0 (0.0%)	
**Thrombocytopenia**	19 (20.2%)	9 (9.6%)	0.041	4 (4.3%)	1 (1.1%)	0.365
**Nausea**	20 (21.3%)	18 (19.1%)	0.716	0 (0.0%)	1 (1.1%)	0.317
**Anorexia**	26 (27.7%)	18 (19.1%)	0.168	1 (1.1%)	2 (2.1%)	1.0
**Diarrhea**	5 (5.3%)	8 (8.5%)	0.388	0 (0.0%)	0 (0.0%)	
**Vomiting**	12 (12.8%)	12 (12.8%)	1.0	1 (1.1%)	2 (2.1%)	1.0
**Fatigue**	24 (25.5%)	20 (21.3%)	0.491	0 (0.0%)	0 (0.0%)	

### Initial Patterns of Failure in Patients With Locally Advanced Gastric Cancer After D2 Dissection

**Figure 1:**
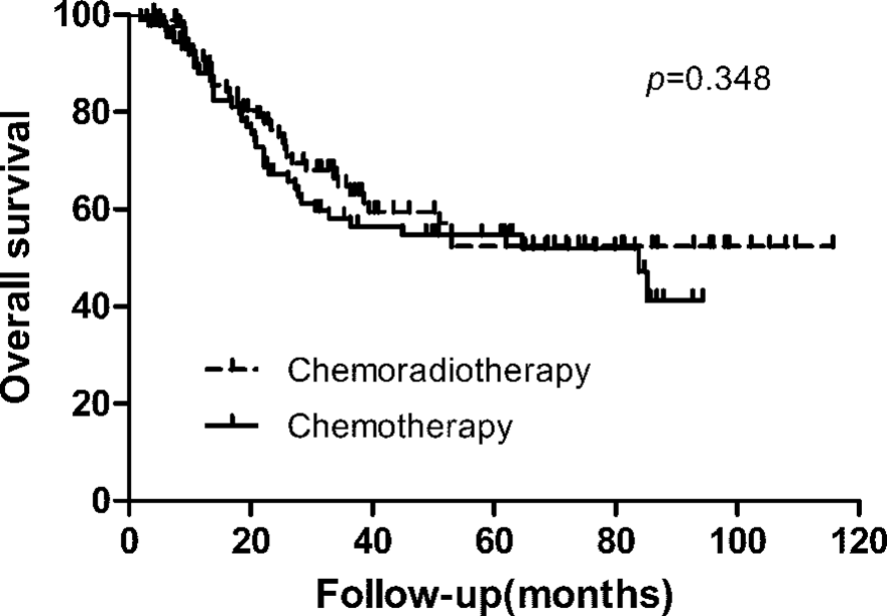
Overall survival.

With a median follow-up of 27.10 months (range, 2–116 months), 79 patients (42.0%) of 188 patients developed first recurrence events (36 in CRT group and 43 in CT group), including LRR (n = 38), distant metastasis (n = 37), and peritoneal dissemination (n = 35). The most common site of LRR was regional nodes recurrence (7 in CRT group and 17 in CT group). The estimated 3-year DFS and overall survival in the CRT and CT groups was 59.3% vs. 50.9% (*p* = 0.239), and 64.8% vs. 58.1% (*p* = 0.348, **Figure 1**), respectively. We also analyzed the first site of recurrence, patients with LRR alone showed the great benefit from CRT (4.3% vs. 11.7%, *p* = 0.038, **Table 3**). However, with or without LRR, there was barely no difference in patients with distant or peritoneal metastasis (**Table 3**). In CRT group, two of four patients with LRR alone experienced infield failure and another two patients experienced out of field failure.

**Table 3:** First site of recurrence in CRT and CT group.

	CT, n = 94	CRT, n = 94	*p*-value
**LRR only, n(%)**	11 (11.7)	4 (4.3)	0.038
**LRR and distant or peritoneum, n(%)**	13 (13.8)	10 (10.6)	0.359
**Distant or peritoneum only, n(%)**	19 (20.2)	22 (23.4)	0.922

Eight patients experienced both distant metastasis and peritoneal dissemination.

**Table 4** has shown the initial patterns of failure in different N stages between these two groups. The CT group showed an increased risk of LRR compared to the CRT group (25.5% *vs.* 14.9%, *p* = 0.044), while there were no statistically differences in other sites (peritoneal dissemination, distant metastasis) between these two groups. In patients with N1–3a stage, LRR was significantly deceased after receiving additional radiation (25.4% *vs.* 14.1%, *p* = 0.046), while there was no obvious difference between the N3b subgroups (31.6% *vs.* 21.1%, *p* = 0.826).

**Table 4:** Patterns of failure in CRT and CT group.

	Total			N1–3a			N3b		
	CT, n = 94	CRT, n = 94	*p*-value	CT, n = 71	CRT, n = 71	*p*-value	CT, n = 19	CRT, n = 19	*p*-value
**Relapse, n(%)**	43 (45.7)	36 (38.3)	0.184	34 (47.9)	24 (33.8)	0.050	9 (47.3)	11 (57.9)	0.435
**Local/regional, n(%)**	24 (25.5)	14 (14.9)	0.044	18 (25.4)	10 (14.1)	0.046	6 (31.6)	4 (21.1)	0.826
**Peritoneum, n(%)**	17 (18.1)	18 (19.1)	0.849	13 (18.3)	12 (16.9)	0.524	4 (21.1)	6 (31.6)	0.246
**Distant, n(%)**	18 (19.1)	19 (20.2)	0.865	15 (21.1)	10 (14.1)	0.161	3 (15.8)	8 (42.1)	0.121

### Pattern of Para-Aortic Nodes Recurrence

Para-aortic lymph node (station No. 16) was the most frequent site of LRR (7/14 in CRT group and 12/24 in CT group). The sites of para-aortic lymph nodes recurrence were a1 (n = 5/94, 5.3%), a2 (n = 3/94, 3.2%), b1 (n = 3/94, 3.2%), b2 (n = 1/94, 1.1%) in CRT group and a1 (n = 3/94, 3.2%), a2 (n = 11/94, 11.7%), b1 (n = 10/94, 10.6%), b2 (n = 2/94, 2.1%) in CT group. After receiving radiotherapy, recurrence of 16 a2 station and 16 b1 station were significantly deceased (*p* = 0.026, 0.044, respectively, while there was no obvious difference in 16 a1 and 16 b2 stations (*p*>0.05). The estimated 3-year para-aortic nodes recurrence free survival in the CRT and CT groups was 90.7% *vs.* 81.2% (*p* = 0.087).

### Estimation of the Time of Adjuvant Chemoradiotherapy

During the adjuvant CRT periods, the median time from surgery to adjuvant radiotherapy were 3.7 months (range, 1.0–10.0 months) in CRT patients. We grouped patients according to the median time from surgery to adjuvant radiotherapy (<4 moths and ≥4 moths). 60 patients who received irradiation within 4 months after surgery showed a decreased risk of 3-year LRR compared to 34 patients ≥4 months after surgery (6.7% *vs.* 26.5%, *p* = 0.022), while there were no statistically differences in distant metastasis or peritoneal dissemination between the two groups.

## Discussion

Efforts to improve treatment results of locally advanced gastric cancer patients with surgery mainly include use of drugs and radiotherapy. Intergroup study 0116 has shown improvement of the survival with postoperative CRT (11, 12). However, a significant criticism of this trial was the limited extent of the surgical procedure. The ARTIST trial (13) failed to demonstrate any significant differences in DFS between CT and CRT groups after D2 dissection. There was a large proportion of patients with early stage disease enrolled in the trial, who would be the least likely to benefit from RT. In the ARTIST 2 trial (14), there was no significant benefit for the addition of RT to SOX in terms of DFS. However, we need to wait for the detailed data to be republished. In the Dutch CRITICS trial, there were no significant differences in 5-year overall survival or DFS. However, only approximately one half of the patients in both groups could complete the full postoperative treatment (15). Thus, the available data on whether there is a benefit from postoperative radiotherapy in patients receiving radical D2 gastrectomy is still controversial. For all of these reasons, the interest of presented study is to select patients who can benefit from radiotherapy and the suitable radiation target volume and time of adjuvant radiotherapy.

Our study confirms that adjuvant CRT significantly improves local control compared to CT alone. No differences in DFS were observed between CT and CRT groups, which might be due to the risk of peritoneal and distant metastasis. One problem in designing prospective trials of adjuvant RT is the need to select patients most likely to benefit from improved local control. According to current studies, LRR was significantly lower in patients who received CRT (13%–24.2% in CT patients and 7%–15.6% in CRT patients) (13, 16–18). In our study, there is a decreased LRR rate in the CRT group compared with that in the CT group (14.9% *vs.* 25.5%, *p* = 0.044) and this difference highlights the importance of CRT after D2 dissection.

In this study, all patients were treated with IMRT in the CRT group. The acute toxicity during CRT was mild. Only 8.5% patients had grade 3 to 4 toxicities (including neutropenia and thrombocytopenia), but this did not translate into febrile neutropenia or bleeding, and was therefore deemed clinically irrelevant by patients**’** treating physicians. In the CRITICS study, patients were treated by 3D conformal or IMRT techniques (19). Treatment-related serious adverse events were less frequent in our study compared with that in the CRITICS study (8.5% *vs.* 16%). This might be because the IMRT decreased the toxicity.

Subgroup analysis of ARTIST trial revealed an improved DFS after radiation for patients with positive lymph nodes (13). However, the optimal regimen is still not established. Our previous study showed that pN3b patients would suffer earlier distant metastasis or peritoneal dissemination than patients with pN1-3a (20). In subgroup analyses of our recent study, though all the patients received adjuvant CT in the CT group, the rate of LRR (25.5%) remained high. Our study discovered that the pN1-3a patients (25.4% *vs.* 14.1%, *p* = 0.046) benefit more from additional irradiation than patients with pN3b (31.6% *vs.* 21.1%, *p* = 0.826) in reducing LRR. In patients with more than 15 lymph nodes metastases (pN3b stage), the probability of LRR, peritoneal and distant metastases were comparable between both groups (*p*>0.05), leading to a limited benefit from CRT. These results provide support for adjuvant CRT in pN1-3a patients with D2-resected gastric cancer who may benefit from local treatment.

Para-aortic lymph node is the most frequent site of regional nodes recurrence in patients with D2-resected gastric cancer (21). Consistent with other published reports (22), our previous study also found the incidence of para-aortic lymph nodes recurrence was high (74.7% in regional nodes recurrence) (20). However, para-aortic lymph node region is not recommended for prophylactic irradiation according to the National Comprehensive Cancer Network (NCCN) guideline (6). In the present study, the para-aortic lymph nodes areas (16 a1, a2, b1) are irradiated in the CRT group. Our results shows that adding of RT to adjuvant CT significantly deceases the risk of developing recurrence of a2 and b1 region (*p* = 0.026 and *p* = 0.044, respectively), which suggests that LAGC patients might benefit from adjuvant CRT of encompassing para-aortic lymph node. However, this finding is still needed to be confirmed in prospective trials.

The time interval from surgery to radiotherapy may have an effect on the local recurrence rate in patients with gastric cancer. Huang et al. (23) reported 286 patients with stage II to III gastric cancer who underwent curative gastrectomy and adjuvant therapy (CT or CT with concurrent radiotherapy) after 1:1 propensity score matching. The 5-year RFS rates were 57.6% and 46.4% (*p* = 0.028), and DMFS rates were 64.4% and 52.0% (*p* = 0.040) in the early group (≤8 weeks) and the late group (>8 weeks). In our study, patients who received irradiation more than 4 months after surgery showed an increased risk of LRR (*p* = 0.022). Therefore, starting radiation therapy as soon as possible after surgery, especially within the first 4 months after surgery, is suggested. In practice, we prefer to select patients with pre- and post-operative CT. At 4 months after surgery, they normally finished adjuvant CT, and then starting radiotherapy will not influence the tolerance of adjuvant CT.

The limitation of the study is that this is a small retrospective study although eligible patients underwent propensity score matching, with no significant imbalances. It is less robust than prospective randomized trials. Another limitation is the short follow-up time (average follow-up, 27.10 months). Whether the better local control can be translated into survival benefits is still not clear.

## Conclusions

The results of this retrospective study using propensity score matching demonstrate that adjuvant CRT significantly reduced local regional recurrence after D2 dissection of locally advanced gastric cancer. And it is recommended in patients with N1-3a stage rather than N3b stage within 4 months after surgery. It is also recommended that the CTV include a2 and b1 regions of para-aortic lymph nodes. The optimal regimens for CRT should be evaluated in future studies.

## Data Availability Statement

The original contributions presented in the study are included in the article/supplementary material. Further inquiries can be directed to the corresponding author.

## Author Contributions

S-BW conducted data extraction, quality appraisal, data synthesis and analysis, and drafted the manuscript. W-XQ and W-GC designed the protocol, performed the search, data extraction, quality appraisal, data synthesis and interpretation, and drafted the manuscript. J-YC and CX contributed to writing and editing the manuscript. LC and RC determined the scope of the review and contributed to protocol design and writing and editing the manuscript. GC had full access to the data, takes responsibility for data integrity, and is the guarantor of the review. All authors contributed to the article and approved the submitted version.

## Funding

This study was funded by the National Natural Science Foundation of China (grant number 81803164, 81673102, 81602791, 81972963).

## Conflict of Interest

The authors declare that the research was conducted in the absence of any commercial or financial relationships that could be construed as a potential conflict of interest.
